# Inactivation of *Salmonella* Typhimurium by Non-Thermal Plasma Bubbles: Exploring the Key Reactive Species and the Influence of Organic Matter

**DOI:** 10.3390/foods9111689

**Published:** 2020-11-18

**Authors:** Ki Ho Baek, Ye Seul Heo, Joo Young Park, Taemin Kang, Yee Eun Lee, Junghyun Lim, Seong Bong Kim, Cheorun Jo

**Affiliations:** 1 Department of Agricultural Biotechnology, Center for Food and Bioconvergence and Research Institute of Agriculture and Life Sciences, Seoul National University, Seoul 08826, Korea; kihoback@naver.com (K.H.B.); dptmf_1@naver.com (Y.S.H.); British3616@naver.com (T.K.); esdel96@snu.ac.kr (Y.E.L.); 2 Surface Technology Division, Korea Institute of Material Sciences (KIMS), Changwon 51508, Korea; jypark@kims.re.kr; 3 Plasma Technology Research Center of National Fusion Research Institute, 37 Dongjangsan-ro, Gunsan-si, Jeollabuk-do 54004, Korea; limjh@nfri.re.kr (J.L.); sbkim@nfri.re.kr (S.B.K.)

**Keywords:** plasma-activated water, *Salmonella* Typhimurium, ozone, singlet oxygen, superoxide anion radical, organic matter

## Abstract

The key reactive species generated by non-thermal plasma bubbles for the inactivation of *Salmonella* Typhimurium and the effects of organic matter on the inactivation efficacy were investigated. Plasma, which is primarily composed of ozone (O_3_), was generated by dielectric barrier discharge and injected into a solution (400 mL) as a bubble. The surviving population of *S*. Typhimurium decreased in proportion to the treatment time, resulting in a 5.29 log reduction after 5 min of treatment. Verification tests to specify key reactive species were conducted using an O_3_ destruction unit and reactive oxygen species scavengers. The results indicated that singlet oxygen (^1^O_2_) contributes substantially to the inactivation of *S*. Typhimurium, and that the presence of superoxide anion radicals (O_2_**^·^**^−^) from O_3_ is essential for the production of ^1^O_2_. When a *S*. Typhimurium suspension containing organic matter (final concentration: 0, 0.005, 0.05, 0.1, and 0.5 g/L), consisting of beef extract and peptone, was treated with plasma bubbles for 5, 10, 15, 20, 25, and 30 min, respectively, the potential of the plasma bubbles for inactivating *S*. Typhimurium successfully was verified with longer contact time, despite organic matter attenuating the inactivation efficiency in a dose-dependent manner.

## 1. Introduction

As the food industry continues to develop, consumers are demanding safer, fresher, and higher-quality foods [1]. However, despite technological advances, food products can still become contaminated with pathogenic bacteria at any point from production to final consumption, including farming, harvesting, processing, transportation, and storage, which can cause serious health problems and global economic losses [2,3]. Therefore, it is necessary to develop a technology that can effectively inactivate pathogenic microorganisms without compromising food quality. In this regard, it is important to prevent food spoilage in advance and reduce the risk of food poisoning through proper cleaning and sanitization [4]. Many previous studies have been conducted to develop effective sanitizers and disinfectants by utilizing various substances, including chlorine-containing chemicals [5,6], electrolyzed water [7,8], organic acid [9,10,11], ozone (O_3_) [12,13], and plasma-activated solutions [14,15,16], and studies have verified that these substances can effectively inactivate microorganisms. Among the various disinfectants, plasma-activated water (PAW) has attracted significant attention due to its properties, which include virus inactivation, wound healing, the promotion of plant growth, and microbial inactivation [17].

PAW, which is easy to operate, safe, and highly efficient [11], is usually produced by the treatment of non-thermal (non-equilibrium, cold) plasma in water. Various reactive molecules, commonly called reactive oxygen and nitrogen species (RONS), can be formed by gas-phase discharges and can dissolve or penetrate the liquid (at the gas–liquid interface) to produce secondary reactive species [18]. Secondary aqueous RONS, such as hydroxyl radicals (·OH), hydrogen peroxide (H_2_O_2_), singlet oxygen (^1^O_2_), superoxide anion/perhydroxyl radicals (O_2_**^·^**^−^/HO_2_**^·^**), O_3_, nitrite/nitrate (NO_2_^−^/NO_3_^−^) and peroxynitrites/peroxynitrous acid (ONOO^−^/ONOOH), can initiate chemical and biocidal processes in the liquid [19]. Each reactive species has a different oxidative potential and lifetime [20], and the type and concentration of the reactive species produced in the liquid depend on the discharge conditions, gas types, solution types, etc. [17]. In this regard, PAW can be considered in two aspects, depending on the application method: (1) short- and long-lived reactive species, which are generated in the liquid at the same time as plasma treatment, react with the target in real-time, and (2) short- and long-lived reactive species, which are produced and remain in the liquid during plasma treatment (mainly long-lived reactive species) and newly-generated by post-discharge reactions after plasma treatment, are used separately to react with the target. In fact, Ma et al. demonstrated that short-lived reactive species can also be subsequently produced after discharge through the following reactions (Reactions (1)–(5)) [21]. Therefore, it is important to consider appropriate PAW generation and application methods, depending on the purpose.

ONOOH + H_2_O_2_ → O_2_NOOH + H_2_O (1)

O_2_NOOH → O_2_NOO^−^ (2)

O_2_NOO^−^ → ^1^O_2_ + NO_2_^−^ (3)

O_2_NOO^−^ → NO_2_ + O_2_^−^ (4)

2O_2_^−^ + 2H^+^ → ^1^O_2_ + H_2_O_2_ (5)

Since most reactive species have a significantly short half-life and can react with a variety of organic substances (Table 1) [22], there are some possible limitations to the effective pasteurization of bacteria present on food surfaces or in solutions containing organic matter [4], as can be found in other sanitizers or disinfectants [23,24]. To overcome some of these limitations, previous studies have aimed to improve the efficiency of plasma treatment for solutions by incorporating plasma into various technologies [25,26,27], one of which involves using bubbles as a potential means of improving the efficiency of mass transfer [28,29]. However, few studies have revealed the clear pasteurization mechanism of plasma bubbles.

In this study, a non-thermal plasma bubble was applied for the direct plasma treatment of liquid, and *S.* Typhimurium was used as a model pathogenic bacterium. The inactivation efficacy of plasma bubbles against *S.* Typhimurium was examined, and the mechanism of its bactericidal action was investigated by exploring the key reactive species in PAW. In addition, the effects of organic matter on the *S*. Typhimurium inactivation efficiency of plasma bubbles were investigated.

## 2. Materials and Methods

### 2.1. Plasma Generator and Plasma-Bubbling System

**Figure 1:**
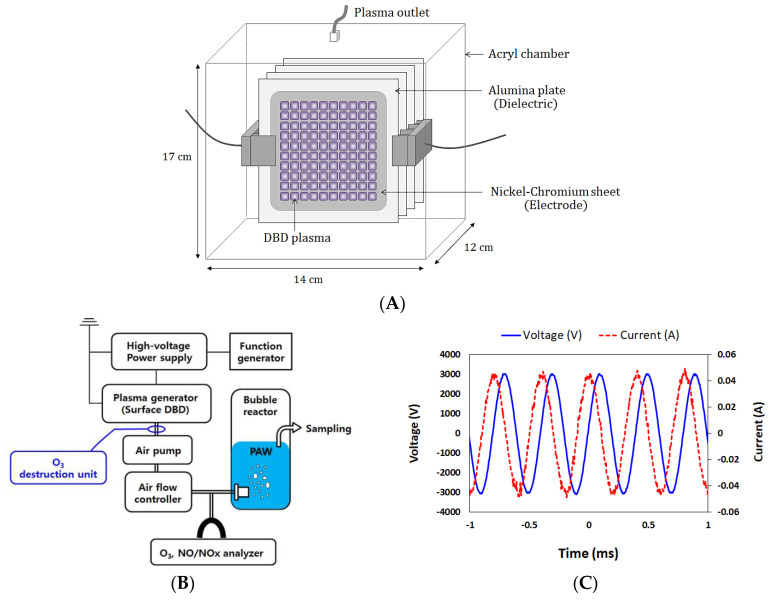
Schematic diagram of (**A**) a dielectric barrier discharge (DBD) plasma generator; (**B**) a plasma-bubbling system (O_3_ destruction unit was optionally applied according to experimental conditions); and (**C**) voltage and current waveforms during discharge. PAW: plasma-activated water.

Figure 1 shows a schematic diagram of the dielectric barrier discharge (DBD) plasma generator and plasma-bubbling system used in this study. The DBD apparatus consisted of four aluminum oxide plates (100 × 100 × 0.635 mm) with two nickel–chromium sheets attached back and forth as electrodes (Figure 1A). The major components of the plasma bubbling system included a function generator (Agilent 33500B series; Agilent Technologies, Loveland, CO, USA), high-voltage amplifier (model 5/80; Trek, Inc., Lockport, NY, USA), plasma generator, air pump, air flow controller, and bubbler (SL-03; Sang-A Pneumatic Co., Ltd., Daegu, Korea) (Figure 1B).

### 2.2. The Generation of Plasma Bubbles

Table 2 shows the operating conditions for generating the plasma bubbles used in this study. Plasma was generated between two electrodes separated by an aluminum oxide plate (Figure 1A) and was injected into deionized water (400 mL) at a gas flow rate of 4 L/min through a bubbler. The specific voltage and current waveform generated from the DBD are presented in Figure 1C. Voltage and current profiles were obtained using a digital oscilloscope (DPO 2024, Tektronix, Beaverton, OR, USA) equipped with a voltage probe (P6015A, Tektronix, Beaverton, OR, USA) and current probe (Pearson 411, Pearson Electronics, Inc., Pal Alto, CA, USA).

### 2.3. Measurement of Gaseous O_3_ and Nitrogen Oxides (NO/NOx)

To measure the actual amount of gaseous reactive species injected into the bubble reactor, the gas generated under the same operating conditions was collected and analyzed just before the gas was injected into the bubble reactor. The concentrations of gaseous O_3_ and NO/NOx were measured using an O_3_ analyzer (106-M, 2B Technologies, Boulder, CO, USA) and NO/NOx gas analyzer (nCLD 63, Eco Physics AG, Duernten, Switzerland), respectively.

### 2.4. Chemical Measurement in PAW

The chemical properties of PAW were analyzed simultaneously with plasma bubble treatment (0–5 min). Temperature and pH were measured using a thermometer (YF-160 Type-K, YFE, Hsinchu City, Taiwan) and pH meter (SevenGo, Mettler-Toledo International Inc., Schwerzenbach, Switzerland), respectively. The concentration of dissolved O_3_ was analyzed using the indigo method (standard method 4500-O_3_ B) [30]. NO_2_^−^ and NO_3_^−^ were analyzed using test kits (TNT840, HACH Co., Loveland, CO, USA) and HACH Test ‘N Tube Reactor/Cuvette Tubes with NItraVer X Reagent (Chromotropic Acid method), respectively. A spectrophotometer (DR 1900, HACH Co., Loveland, CO, USA) was used to measure NO_2_^−^ and NO_3_^−^. The concentration of ^1^O_2_ in PAW (0 and 5 min, respectively) was measured using an electron spin resonance spectroscope (JES-X320, Jeol Ltd., Tokyo, Japan). A final concentration of 10 mM 2,2,6,6-tetramethylpiperidine (TEMP) was used as a spin trap reagent for trapping ^1^O_2_. In addition, to verify the contribution of superoxide anions (O_2_**^·^**^−^) to the production of ^1^O_2_, the concentration of ^1^O_2_ in PAW containing 10 mM tiron (a scavenger of O_2_**^·^**^−^) was also analyzed. All scans were carried out with the following instrument settings: sweep range, 7.5 mT; microwave power, 1 mW; modulation width, 0.3 mT; time constant, 0.03 s; central magnetic field, 336 mT; scanning time, 30 s. Each sample was measured three times and averaged. Hyperfine coupling constants of the signal were obtained using isotropic simulation software (Jeol Ltd., Tokyo, Japan).

### 2.5. Bacterial Strain and Culture Conditions

The Gram-negative bacterium *S*. Typhimurium ATCC (American Type Culture Collection) 13311 used in this study was provided by the Korean Culture Center of Microorganisms (Seoul, Korea). *S*. Typhimurium was cultivated in fresh sterile nutrient broth (NB; Difco, Becton Dickinson Co., Sparks, MD, USA) at 37 °C with 120 rpm orbital agitation for 24 h. The cells were washed twice with sterile 0.85% saline solution by centrifugation at 2265× *g* for 15 min at 4 °C using a refrigerated centrifuge (UNION 32R, Hanil Science Industrial Co. Ltd., Gimpo, Korea). The pellets were re-suspended in sterile 0.85% saline solution at a final concentration of 10^8^ to 10^9^ colony-forming units (CFU)/mL.

### 2.6. Plasma Bubble Treatment of the Bacterial Suspension and Assessment of Bacterial Inactivation

For the bacterial inactivation assay, sterile deionized water was inoculated by adding a prepared bacterial suspension to a final concentration of 10^6^–10^7^ CFU/mL. Then, 400 mL of bacterial suspension was treated with plasma bubbles for 1, 2, 3, 4, and 5 min. After, NB (Difco) consisting of 37.5% beef extract and 62.5% peptone by weight was used to evaluate the effect of organic matter on the inactivation efficacy of plasma bubbles against *S*. Typhimurium suspension. Different final concentrations (0, 0.005, 0.05, 0.1, and 0.5 g/L) of NB-containing bacteria were treated with plasma bubbles for 5, 10, 15, 20, 25, and 30 min, respectively. After treatment, an aliquot (1 mL) of the sample was immediately transferred to 9 mL Dey–Engley neutralizing broth (Difco), mixed well, and decimally diluted. To enumerate both uninjured and injured *S*. Typhimurium, the thin agar layer (TAL) method [31] was applied to recover injured cells. The selective and non-selective media used for TAL were xylose lysine deoxycholate agar (Difco) and nutrient agar (Difco), respectively. To prepare TAL plates, solidified selective medium was overlaid with 7 mL of melted non-selective medium (48 °C), and another 7 mL of melted non-selective medium was overlaid (7 + 7 mL; two times overlay) after solidification of the first layer. Each diluent (100 μL) was plated onto the TAL plates and incubated at 37 °C for 24 h.

### 2.7. Application of Reactive Oxygen Species (ROS) Scavengers and O_3_ Destruction Unit

To evaluate the roles of ·OH, H_2_O_2_, ^1^O_2_, and O_2_**^·^**^−^ in the bactericidal action of plasma bubbles, a final concentration of 200 mM d-mannitol (·OH scavenger), 10 mM sodium pyruvate (H_2_O_2_ scavenger), 5 mM sodium azide (^1^O_2_ scavenger), 10 mM l-histidine (^1^O_2_ scavenger), or 10 mM tiron (O_2_**^·^**^−^ scavenger) was added to the bacterial suspension before plasma bubble treatment. The types and concentrations of these scavengers have already been proven to be effective through several previous studies [32,33,34]. The bacterial suspension with or without scavengers was treated with plasma bubbles for 5 min, and the inactivation rates were determined as described above. In addition, dissolved O_3_ analysis and bacterial inactivation assays were conducted after artificially removing O_3_ gas using an O_3_ destruction unit (ODS-2P, Ozone Solutions, Hull, IA, USA), which can remove up to 1% of O_3_ gas, to verify its contribution to the antibacterial action of plasma bubbles.

### 2.8. Statistical Analysis

All experiments were conducted in triplicate. SAS statistical software (version 9.4, SAS Institute Inc., Cary, NC, USA) was used to analyze the data. Student’s *t*-test and Tukey’s multiple comparison test were used for statistical analyses. Significant differences among the mean values were established at a significance level of *p* < 0.05.

## 3. Results

### 3.1. Physicochemical Characterization of Plasma

**Figure 2:**
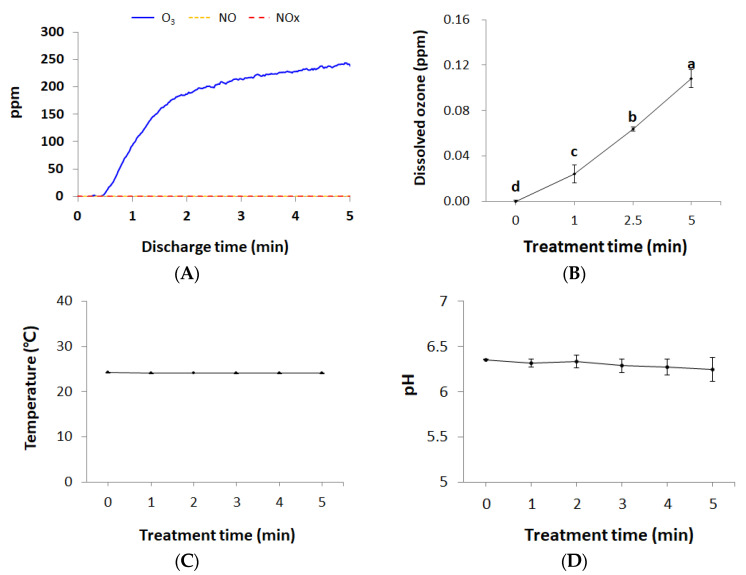
(**A**) Gaseous O_3_ and NO/NOx concentrations during discharge; (**B**) dissolved O_3_ in PAW; (**C**) temperature and (**D**) pH value of PAW according to the plasma bubble treatment time. Error bars denote standard deviation. ^a,b,c,d^ Different letters indicate significant differences (*p* < 0.05).

During discharge, gaseous O_3_ continuously increased from 0 to 240 ppm over 5 min, whereas NO/NOx did not occur (detection limit: 0.05 ppm) (Figure 2A). As a result, the dissolved O_3_ concentration in PAW increased with treatment time, and was 0.11 ± 0.008 ppm after 5 min treatment (Figure 2B). On the other hand, after 5 min of plasma bubble treatment, the concentration of NO_2_^−^ in PAW was below the detection limit (2.00 ppm) (Figure S1A), and the concentration of NO_3_^−^ was only 1.39 ± 0.051 ppm (Figure S1B). During plasma bubble treatment, the liquid temperature and pH remained at 24.12 ± 0.032 °C (Figure 2C) and 6.30 ± 0.042 (Figure 2D), respectively, until 5 min. For atmospheric air discharge [21,35], the pH of the liquid generally decreased during plasma treatment due to the production of HNO_2_ and HNO_3_. These molecules release hydrogen ions through deprotonation reactions, thus acidifying water. However, the current DBD condition did not generate enough NOx to drastically lower the pH of the water within 5 min (Figure 2A,D).

### 3.2. Bactericidal Effects of Plasma Bubbles and Contributions of Reactive Species

**Figure 3:**
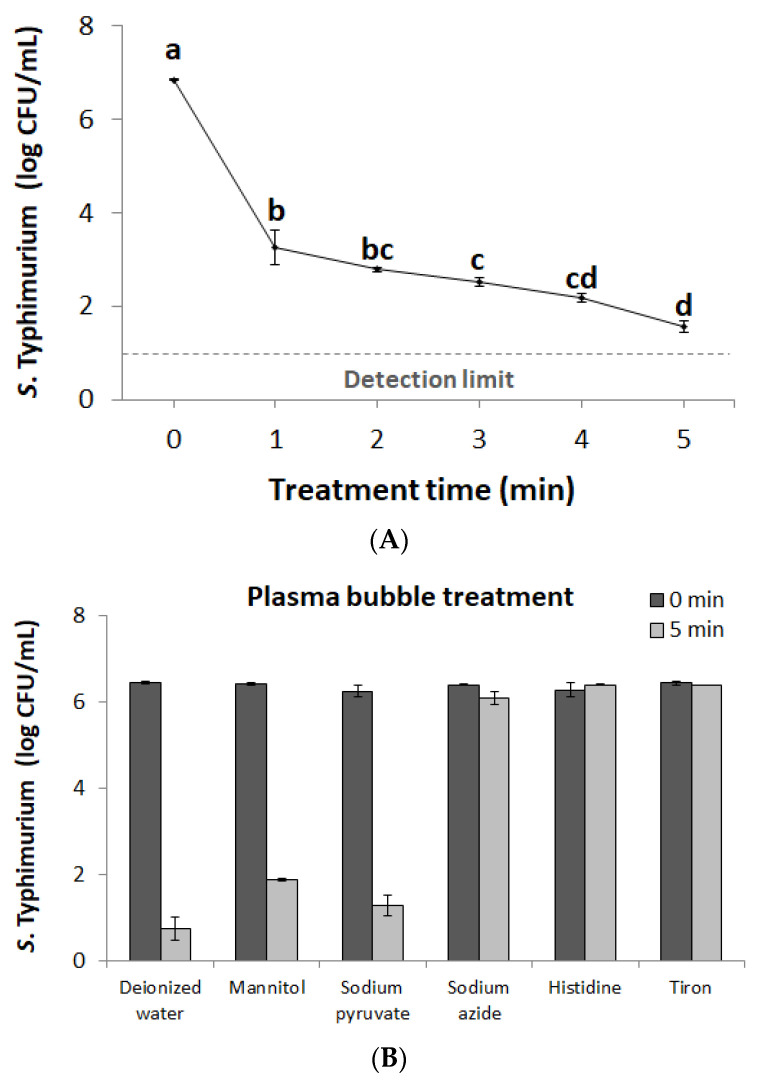
(**A**) Surviving population (log colony-forming units (CFU)/mL) of *S*. Typhimurium after different plasma bubble treatment times (0, 1, 2, 3, 4, and 5 min) and (**B**) *S*. Typhimurium inactivation results by 5 min plasma bubble treatment with the addition of different reactive oxygen species (ROS) scavengers (d-mannitol for ·OH; sodium pyruvate for H_2_O_2_; sodium azide and l-histidine for ^1^O_2_; tiron for O_2_**^·^**^−^). Error bars denote standard deviation. ^a,b,c,d^ Different letters indicate significant differences (*p* < 0.05).

The inactivation efficacy of plasma bubbles against *S*. Typhimurium is depicted in Figure 3A. The number of viable cells decreased significantly in proportion with the treatment time (*p* < 0.05). The results indicate that plasma bubble treatment effectively inactivated *S*. Typhimurium floating in the solution, and that certain reactive species produced by plasma bubbles contributed to bactericidal action. At this time, ROS should be considered more important than reactive nitrogen species because it has already been verified that the generation and contribution of NOx may be negligible through the results of gaseous NO/NOx concentrations and solutions pH (Figure 2). Therefore, a series of ROS scavengers, such as d-mannitol for ·OH, sodium pyruvate for H_2_O_2_, sodium azide and l-histidine for ^1^O_2_, and tiron for O_2_**^·^**^−^ were used to evaluate the possible roles of plasma bubbles in the bacterial inactivation process (Figure 3B). As a result of the experiment, sodium azide, l-histidine, and tiron almost completely eliminated the bactericidal effects of plasma bubbles. These data suggest that ^1^O_2_ and O_2_**^·^**^−^ were the main functional species of the plasma bubble in the inactivation of *S*. Typhimurium.

### 3.3. The Role of Gaseous O_3_ on the Bactericidal Action of Plasma Bubbles

**Figure 4:**
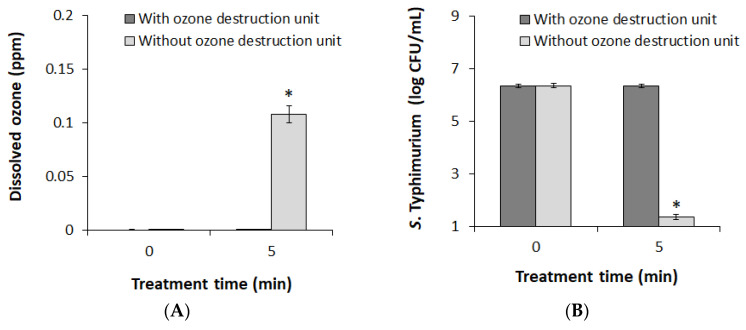
(**A**) Concentrations of dissolved O_3_ in PAW; (**B**) the surviving population (log CFU/mL) of *S*. Typhimurium after 5 min of plasma bubble treatment according to O_3_ filtration. Error bars denote standard deviation. Student’s *t*-test; * *p* < 0.05 with respect to the untreated control.

Under the DBD conditions applied in this study, gaseous O_3_ was produced the most and was injected into the water as a bubble (Figure 2A,B). In the present study, the reactive species contributing most to the bactericidal action of plasma bubbles were ^1^O_2_ and O_2_**^·^**^−^ (Figure 3B). Hence, the first consideration was to verify the relationship between gaseous O_3_ and the key reactive species (^1^O_2_ and O_2_**^·^**^−^). The O_3_ destruction unit was used to artificially eliminate O_3_ gas produced from plasma generators, and the concentration of dissolved O_3_ in solution was measured to assess whether O_3_ gas was completely removed. Figure 4A shows the dissolved O_3_ concentration in the solution according to the O_3_ filtration, which confirmed that there was no dissolved O_3_ in the water under the O_3_ gas destruction unit during the 5 min treatment of plasma bubbles. The results of applying the same conditions to *S*. Typhimurium inactivation experiments are presented in Figure 4B. Since the filtration of gaseous O_3_ completely eliminated the bactericidal action of plasma bubbles against *S*. Typhimurium for 5 min of treatment (Figure 4B), it was confirmed that the production of O_3_ gas and injection into the solution must precede the inactivation of *S*. Typhimurium by plasma bubbles.

### 3.4. Generation of Key Reactive Species by Plasma Bubbles

**Figure 5:**
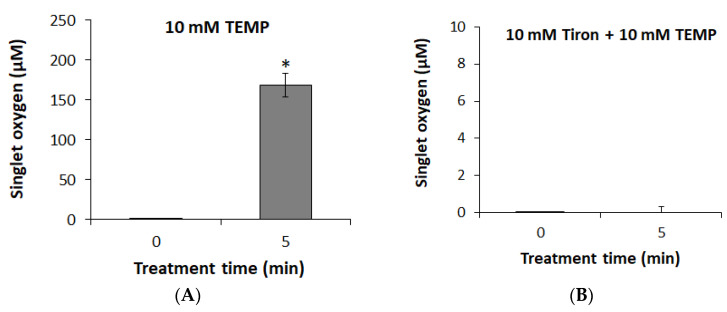
Concentrations of accumulated ^1^O_2_ in PAW (**A**) without or (**B**) with the addition of 10 mM tiron (a scavenger of O_2_**^·^**^−^) during 5 min plasma bubble treatment. TEMP: 2,2,6,6-tetramethylpiperidine (a spin trap reagent for trapping ^1^O_2_). Error bars denote standard deviation. Student’s *t*-test; * *p* < 0.05 with respect to the untreated control.

The production of ^1^O_2_ in PAW was analyzed because the role of ^1^O_2_ in the bactericidal action of *S*. Typhimurium by plasma bubbles is important (Figure 3B). The amount of accumulated ^1^O_2_ after 5 min of plasma bubble treatment was approximately 168.40 ± 14.812 µM (Figure 5A). Since it was necessary to identify which reactive species mainly contributed to the formation of ^1^O_2_ during plasma bubble treatment, whether O_2_**^·^**^−^, which plays a role in *S*. Typhimurium inactivation at a level equivalent to ^1^O_2_, could affect the formation of ^1^O_2_ was first considered. After 5 min of plasma bubble treatment with the addition of tiron (a scavenger of O_2_**^·^**^−^), it was confirmed that no ^1^O_2_ was produced in the PAW (Figure 5B). These results indicate that O_2_**^·^**^−^ is fully involved in the final production of ^1^O_2_, which, in turn, has a direct effect on *S*. Typhimurium inactivation.

### 3.5. Effects of Organic Matter on the Inactivation Efficacy of Plasma Bubbles

**Figure 6:**
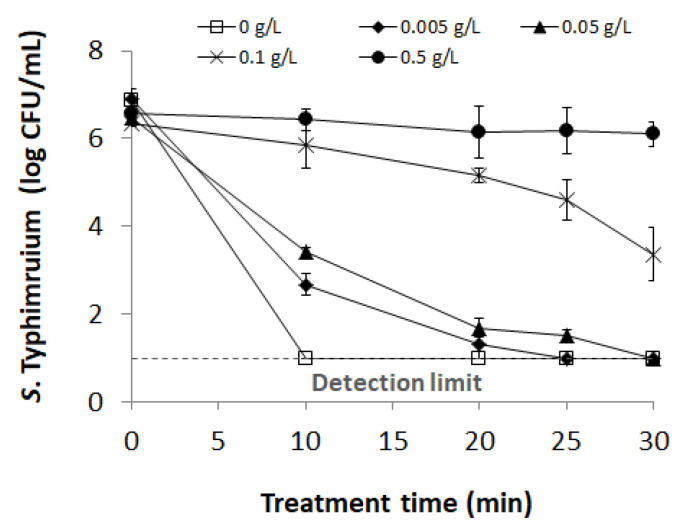
Effects of different concentrations of organic matter (final concentration: 0, 0.005, 0.05, 0.1, and 0.5 g/L) on the inactivation efficacy of plasma bubbles against *S*. Typhimurium according to plasma bubble treatment time (0, 10, 20, 25, and 30 min). The organic matter consisted of 37.5% beef extract and 62.5% peptone by weight. Error bars denote standard deviation.

The effects of organic matter on the inactivation efficacy of plasma bubbles against *S*. Typhimurium are presented in Figure 6. First, plasma bubble treatment in the absence of organic matter effectively inactivated *S*. Typhimurium, resulting in a reduction of more than 5.86 log CFU/mL within 10 min (*p* < 0.05). On the other hand, *S*. Typhimurium inactivation efficiency in organic mixtures (0.005–0.5 g/L) according to plasma bubble treatment time decreased as the concentration of organic matter increased. When plasma bubbles were treated for 10 and 20 min with 0.005 g/L organic matter, the surviving population of *S*. Typhimurium decreased by 4.21 and 5.59 log CFU/mL (*p* < 0.05), respectively, and no surviving bacteria were detected when treated for more than 25 min (detection limit: 10^1^ CFU/mL; Figure 6). The bacterial inactivation efficiency of the plasma bubbles after 10 min of treatment decreased in the 0.05 g/L organic matter compared to 0.005 g/L, but the survival population of *S*. Typhimurium decreased by 3.06 and 4.77 log CFU/mL (*p* < 0.05) as plasma bubbles were treated for 10 and 20 min, respectively, and the bactericidal effect of approximately 5.47 log CFU/mL was shown after 30 min of plasma bubble treatment (*p* < 0.05; Figure 6). However, in organic matter at a 0.1 g/L concentration, the inactivation efficiency of the plasma bubbles for *S*. Typhimurium decreased dramatically, and the 10 min treatment of plasma bubbles did not show any significant bactericidal effect. Under the same conditions, the number of *S*. Typhimurium decreased by 1.17, 1.72, and 2.96 log CFU/mL (*p* < 0.05), respectively, when plasma bubbles were treated for 20, 25, and 30 min (Figure 6). When the concentration of organic matter was 0.5 g/L, the bactericidal effect of the plasma bubbles against *S*. Typhimurium was completely eliminated, and no significant reduction was observed even when the plasma bubbles were treated for up to 30 min (Figure 6). These results indicate that organic matter severely attenuated the inactivation effect of plasma bubbles against *S*. Typhimurium in a dose-dependent manner.

## 4. Discussion

In the current study, DBD plasma was applied to inactivate *S*. Typhimurium in suspension, and high concentrations of O_3_ gases were found to be dominant. At this point, short-lived gas-phase reactive species were excluded, as plasma produced in the plasma generator was injected into the water in a secondary manner. Park et al. reported that the rapidly generated O_3_ at the early stage of discharge was eventually quenched by NO, resulting in the dominance of NO and NO_2_, and that gas temperature (N_2_ vibrational temperature) was one of the important factors in this transition [36]. However, our discharge in air-based surface DBD plasma systems was a condition in which no aforementioned transition occurred, and O_3_ was mainly formed and acted as a major chemical. Therefore, in this study, as the plasma bubble treatment time increased, the concentration of dissolved O_3_ in the water increased, eventually pasteurizing *S*. Typhimurium by more than 5 log CFU/mL within 5 min. Many studies have been conducted involving the application of O_3_ generators for the injection of O_3_ gas into water to inactivate microorganisms or decompose organic matter [13,37,38]. Since O_3_ can directly oxidize various organic substances and microbial components [12], previous studies have suggested that concentrations of gas-phase or dissolved O_3_ in solution are important as oxidative indicators [37,39]. However, not only the concentration of gaseous or aqueous O_3_, but also the specific reactive species that contribute substantially to the degradation of organic matter or inactivation of microorganisms should be considered. Therefore, verification tests were conducted using an O_3_ gas destruction unit and various ROS scavengers, which confirmed that ^1^O_2_ and O_2_**^·^**^−^ were the most important reactive species for the direct inactivation of *S*. Typhimurium in the current system.

^1^O_2_ and O_2_**^·^**^−^ are major ROS and have the potential to improve the bactericidal and virucidal effects of PAW [21,33]. Guo et al. reported that ^1^O_2_ effectively inactivated bacteriophages (double- and single-stranded DNA as well as RNA bacteriophages) in water by attacking both proteins and nucleic acids, resulting in the aggregation of bacteriophages [33]. Ma et al. also proved that ^1^O_2_ and O_2_**^·^**^−^ induced the antibacterial effects of PAW against *Escherichia coli* DH5α, and that both species were generated by post-discharge reactions (Reactions (1)–(5)) due to their short half-life [21]. In order for the aforementioned reactions to occur, sufficiently generated NO_2_^−^ and H_2_O_2_ must react with each other to produce ONOOH, which can further interact with H_2_O_2_ to produce peroxynitric acid (O_2_NOOH). Finally, O_2_NOOH decomposes into ^1^O_2_ and O_2_**^·^**^−^. However, in the current system, it is difficult for ^1^O_2_ or O_2_**^·^**^−^ to be produced through these reactions because NO_2_^-^ is not detected in PAW (detection limit: 2.00 ppm) due to insufficient NOx generation in DBD plasma, and sodium pyruvate (a scavenger of H_2_O_2_) does not eliminate the bactericidal effect of plasma bubbles.

In our plasma bubble system, it was confirmed that the injection of O_3_ gas must precede for generating the source of the antimicrobial action, and the presence of O_2_**^·^**^−^ is necessary for the production of ^1^O_2_. Once O_3_ enters the water, it becomes highly unstable and rapidly decomposes through a complex series of reactions. At this time, hydroxide ions (HO^−^) can initiate a chain reaction, which is sustained by HO_2_**^·^** as follows [40]:

O_3_ + HO^−^ → HO_2_**^·^** + O_2_**^·^**^−^ (6)

The HO_2_**^·^** generated by the above reaction may initiate further reactions and contribute to the production of ^1^O_2_. The possible reactions that have been proposed for converting O_2_**^·^**^−^ to ^1^O_2_ are as follows [41]:

HO_2_**^·^** + HO_2_**^·^** → ^1^O_2_ + H_2_O_2_ (7)

HO_2_**^·^** + O_2_**^·^**^−^ + H^+^ → ^1^O_2_ + H_2_O_2_ (8)

In Reaction (7), two HO_2_**^·^** molecules react together to produce ^1^O_2_ (rate constant of 8.6 × 10^5^ M^−1^s^−1^) [41]. Reaction (8) shows the spontaneous dismutation reaction for superoxide, in which O_2_**^·^**^−^ reacts with HO_2_**^·^** to produce ^1^O_2_ and H_2_O_2_ (rate constant of 9.7 × 10^7^ M^−^^1^s^−^^1^) [41]. As O_2_**^·^**^−^ is essential for the production of ^1^O_2_ in this study, it is worth considering the possibility that the Reaction (8) is the main path for ^1^O_2_ production. In addition, since the solution pH remained at 6.30 ± 0.042 during the 5 min plasma bubble treatment, the reaction rate of Reaction (7) is relatively lower than that of Reaction (8) as the HO_2_**^·^**/O_2_**^·^**^−^ ratio falls 10-fold for each unit rise in pH above a p*K_a_* of 4.8.

As a result of the current study, the presence of organic matter (beef extract and peptone) in water dramatically attenuated the antibacterial efficacy of plasma bubbles against *S*. Typhimurium. Various sanitizers and disinfectants being less effective in the presence of organic matter, including beef extract, peptone, tryptone, and cellulose, has previously been suggested in a number of studies [4,23,24]. Chen et al. demonstrated that organic loads affect the chlorine requirements of produce (romaine lettuce, iceberg lettuce, strawberries, and grapes) wash water, especially as organic loads increase [5]. Jo et al. found that proteins have the greatest negative effect on the antibacterial efficacy of slightly acidic electrolyzed water against *Bacillus cereus* (ATCC 14579; 10987), *Listeria monocytogenes* (ATCC 19118 and Scott A), *E. coli* O157:H7 (ATCC 35150; 43894), and *S. enterica* (*S.* Enteritidis ATCC 13076 and *S*. Typhimurium ATCC 14028) compared to lipids and carbohydrates [24]. Xiang et al. also reported that high concentrations of organic matter affected the physicochemical properties of PAW, such as pH, oxidation-reduction potential (ORP), and NO_2_^−^, thereby reducing the antibacterial properties of PAW [4]. Similarly, in this study, organic matter attenuated the antibacterial property of plasma bubbles in a dose-dependent manner, but PAW is still highly available because longer contact times have allowed it to eventually successfully inactivate microorganisms (except for at the highest organic matter concentrations).

Particularly for PAW, since reactive species have a remarkably short half-life and can react with a variety of organic materials (Table 1) [22], there are some limitations in the pasteurization of microorganisms present on the surfaces of food or in mixture solutions. To overcome some of these limitations, we applied bubble technology, which has the potential to increase the efficiency of mass transfer [28,29], but unfortunately, the characteristics of bubbles (such as bubble size and density) and the efficiency of mass transfer have not yet been considered in depth.

## 5. Conclusions

This study was conducted to determine the key reactive species generated by non-thermal plasma bubbles for the inactivation of *S.* Typhimurium and to examine the effects of organic matter on inactivation efficacy. Plasma bubble treatment effectively inactivated *S*. Typhimurium floating in water, and ^1^O_2_ originating mainly from O_3_ and O_2_**^·^**^−^ contributed substantially to the bactericidal action. Organic matter attenuated the bactericidal action of plasma bubbles in a dose-dependent manner, but the potential for inactivating *S*. Typhimurium was confirmed successfully with longer contact times. In the future, it will be necessary to consider the types and contents of organic matter contained in the target (solid, semi-solid, or liquid phase) for practical applications in food-related materials, and the specific pasteurization mechanism of plasma bubbles through bubble characterization will need to be identified.

## Figures and Tables

**Table 1:** Basic properties of reactive oxygen species in plant tissues (adapted from [22]).

Property	Hydroxyl Radical	Singlet Oxygen	Superoxide	Hydrogen Peroxide
Half-life (in biological system)	1 ns	1 μs	1 μs	1 ms
Penetration depth (diffusion coefficient 10^−9^ m^2^/s)	1 nm	30 nm	30 nm	1 μm
Reacts with:				
Lipids	Rapidly	Polyunsaturated fatty acids	Hardly	Hardly
Carbohydrates	Rapidly	No	No	No
Proteins	Rapidly	Trp, His, Tyr, Met, Cys	Fe-S centers	Cysteines

**Table 2:** Operating conditions for generating plasma bubbles.

Parameter	Conditions
Frequency	2.5 kHz
Peak voltage	3.0 kV
Working gas	Air
Gas flow rate	4 L/min
Electrode composition	Nickel–chromium
Dielectric composition	Aluminum oxide
Bubble reactor composition	Acryl
Sample volume	400 mL

## Supplementary Materials

The following are available online at https://www.mdpi.com/2304-8158/9/11/1689/s1, Figure S1: (A) NO_2_^-^ and (B) NO_3_^-^ concentrations in plasma activated water after 5 min of plasma bubble treatment. Error bars denote standard deviation. Student’s *t*-test; *, *p* < 0.05 with respect to the untreated control.
