# Supplementary material for: Inactivation of Salmonella Typhimurium by Non-Thermal Plasma Bubbles: Exploring the Key Reactive Species and the Influence of Organic Matter
Source: Foods. 2020 Nov 18;9(11):1689. doi: 10.3390/foods9111689 (PMC7698966; doi:10.3390/foods9111689)
Supplement: Supplementary file 1 [file foods-09-01689-s001.pdf]

Supplementary material

Inactivation of *Salmonella* Typhimurium by Non-Thermal Plasma Bubbles: Exploring the Key Reactive Species and the Influence of Organic Matter

Ki Ho Baek, Ye Seul Heo, Joo Young Park, Taemin Kang, Yee Eun Lee, Junghyun Lim, Seong Bong Kim and Cheorun Jo

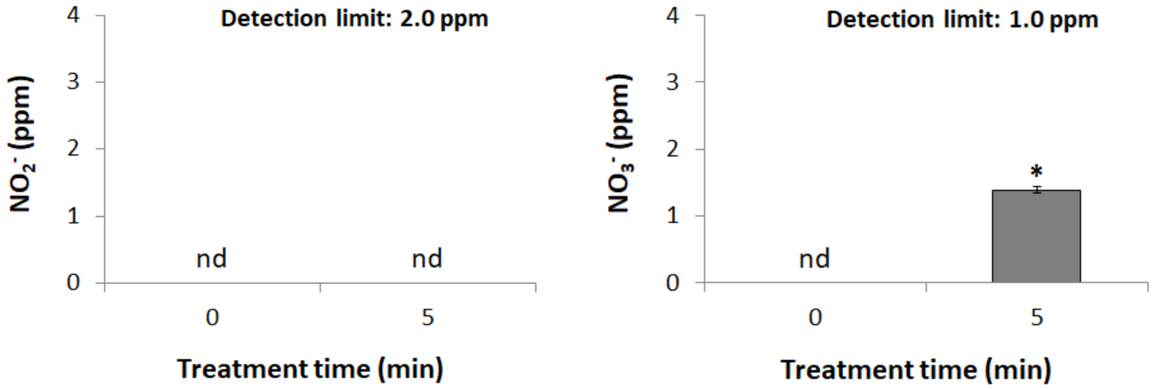

(**A**) (**B**)

Figure S1. (**A**) NO_2^- and (**B**) NO_3^- concentrations in plasma activated water after 5 min of plasma bubble treatment. Error bars denote standard deviation. Student's *t*-test; *, $p < 0.05$ with respect to the untreated control.
